# Ethical challenges for Health Technology Assessment (HTA) in the evolving evidence landscape

**DOI:** 10.1017/S0266462324000394

**Published:** 2024-11-04

**Authors:** Pietro Refolo, Katherine Duthie, Björn Hofmann, Michal Stanak, Neil Bertelsen, Bart Bloemen, Rosella Di Bidino, Wija Oortwijn, Costanza Raimondi, Dario Sacchini, Gert Jan van der Wilt, Kenneth Bond

**Affiliations:** 1 Department of Healthcare Surveillance and Bioethics, Università Cattolica del Sacro Cuore, Rome, Italy; 2 Fondazione Policlinico Universitario A. Gemelli IRCCS, Rome, Italy; 3 John Dossetor Health Ethics Centre, University of Alberta, Edmonton, AB, Canada; 4 Department of Health Science, Norwegian University of Science and Technology, Gjøvik, Norway; 5 National Institute for Value and Technologies in Healthcare (NIHO), Bratislava, Slovak Republic; 6 Health Technology Assessment international (HTAi) Patient & Citizen Involvement, Neil Bertelsen Consulting, Berlin, Germany; 7 Department for Health Evidence, Radboud University Medical Centre, Nijmegen, The Netherlands; 8 Department of Health, Medicine, and Caring Sciences, Linköping University, Linköping, Sweden

**Keywords:** randomized controlled trial, evidence-based medicine, artificial intelligence, ethics

## Abstract

Since its inception, Health Technology Assessment (HTA) has typically determined the value of a technology by collecting information derived from randomized clinical trials (RCTs), in line with the principles of evidence-based medicine (EBM). However, data from RCTs did not constitute the sole source of information, as other types of evidence (such as primary qualitative research) have often been utilized. Recent advances in both generating and collecting other types of evidence are broadening the landscape of evidence, adding complexity to the discussion of “robustness of evidence.” What are the consequences of these recent developments for the methodology and conduct of HTA, the HTA community, and its ethical commitments? The aim of this article is to explore some ethical challenges that are emerging in the current evolving evidence landscape, particularly changes in evidence generation and collection (e.g., diversification of data sources), and shifting standards of evidence in the field of HTA (e.g., increasing acceptability of evidence that is thought of as lower quality). Our conclusion is that deciding how to best maintain trustworthiness is common to all these issues.

## Introduction

Definitions of Health Technology Assessment (HTA) explicitly include the consideration of ethical issues (1;2). A recent international definition defines HTA as “a multidisciplinary process that uses explicit methods to determine the value of a health technology at different points in its lifecycle. The purpose is to inform decision-making in order to promote an equitable, efficient, and high-quality health system” (3). This new definition makes value issues central to HTA.

Ethical questions can be raised with respect to the activity *of* HTA (process) and with respect to issues *in* an HTA (issues raised by a technology) (4–6).

Discussed less often and less explicitly is the fact that HTA also involves an entanglement of facts and values (6-8), and HTA serves a *normative* purpose. This means that empirical inquiry in HTA (i.e., the retrieval, critical examination, and synthesis of evidence to support decision-making) is always linked to ethical (normative) considerations – such as beneficence, nonmaleficence, autonomy, and justice. In other words, practicing HTA means subscribing, usually in a tacit way, to a number of value commitments that guide empirical analysis (8).

Since its inception, HTA has typically determined that value by collecting information derived from randomized clinical trials (RCTs), in line with the principles of evidence-based medicine (EBM). The persistent focus on this kind of evidence has been defined by some as the “stagnation of RCTs” (9). However, data from RCTs did not constitute the sole source of information, as other types of evidence (such as primary qualitative research) have often been utilized. Recent advancements in both generating and collecting other types of evidence are tremendously broadening the landscape of evidence (10).

Technological innovations such as the development of biomarkers, the implementation of digital platforms for sharing data, the introduction of digital endpoints, as well as the emergence of new analytical capabilities (e.g., the so-called “multimodal artificial intelligence”) (11) are altering the design, conduct, and implementation of clinical research. Moreover, electronic health records, mobile apps, wearables, and similar technologies are offering opportunities to collect real-world data (RWD) and generate real-world evidence (RWE). The continuous generation of RWD combined with the collaborative efforts of various organizations for data sharing increases the availability of data tremendously, making it feasible to monitor health technologies across their lifecycle (12).

What are the consequences of these developments for the methodology and conduct of HTA, the HTA community, and its ethical commitments? The new opportunities may add complexity to the discussion of “robustness of evidence” and rekindle and broaden issues that were vigorously debated in the 1980s and 1990s (13). These issues have been recently revisited by some authors in reference to the topic of alternative methods (such as basket trials, platform trials, etc.) for demonstrating causality of outcomes and the value of health interventions (14–17).

Moreover, the increasing call to provide patients with early access to promising new treatments, especially in areas of unmet medical need, pressures regulatory bodies to make decisions with respect to the “strength of evidence” required for marketing approval (13, 18). To enhance early patient access, regulatory and reimbursement processes are becoming increasingly more closely aligned, making appraisal decisions more and more challenging, and putting pressure on HTA bodies. Some of the recommendations or decisions resulting from this process could prove to be ethically problematic.

The following discussion paper aims to explore some ethical challenges that are emerging in this evolving evidence landscape, particularly changes in evidence generation and collection (e.g., diversification of data sources), and shifting standards of evidence in the field of HTA (e.g., increasing acceptability of evidence that is thought of as lower quality according to standard hierarchies of evidence) (19). The article is written from the perspective of individuals and organizations that conduct HTAs (HTA practitioners). The purpose of the article is to identify ethical issues and start to discuss them rather than to propose practical solutions for them.

## Methods

A group of members of the Health Technology Assessment International (HTAi) Interest Group (IG) on Ethical Issues in HTA (https://htai.org/interest-groups/ethics/), including the cochairs of HTAi’s Real-World Evidence & Artificial Intelligence (RWE&AI) IG, met twice during 2023 to discuss the topic. The first meeting was held online on March 9, 2023. One member (PR) prepared and led the discussions, which were based on a review of key documents on both generation of evidence and standards of evidence. The participants critically analyzed the collected material, which was used to identify the main issues for further discussion. Two issues were selected as being of particular interest to the HTA community: the ethical implications of 1) changes in the generation and synthesis of evidence and 2) shifting standards of evidence. A second extended face-to-face meeting was held in Rome, Italy, on March 30–April 1, 2023 to further discuss the development of these issues. The list of participants for both meetings is provided in Supplementary Materials. This article is the output of the discussions, and subsequent writing and revisions were conducted online among all the coauthors.

## Changes in evidence generation and collection

Systematically collecting, evaluating, and summarizing scientific evidence is a key aspect of any HTA. In the traditional practice of HTA, evidence mainly refers to information extracted from published (usually in peer-reviewed journals) articles on RCTs or on a “synthesis” of RCTs (systematic reviews and meta-analyses). However, data from RCTs did not constitute the sole source of information, as other types of evidence (such as primary qualitative research) have often been utilized.

As noted above, there have been significant changes in the dissemination of clinical studies, alongside a transformation in the methods employed for collecting them, as well as in the kind of information that is accessible.

For example, we are witnessing the growth of the number of databases and trial registries that can be accessed: clinical trial registries like ClinicalTrials.gov; complete protocols published in specific journals (like the New England Journal of Medicine); clinical reports on approved drugs provided by regulatory agencies or pharmaceutical companies (e.g., Drugs@FDA); and repositories of clinical reports (e.g., ClinicalStudyDataRequest.com or the Yale University Open Data Access (http://yoda.yale.edu/)). All these have become important sources of information regarding health technologies (20).

Moreover, technological advancement is accelerating the generation of various types of data. The increased use of internet, e-health services, mobile devices, social media, wearable devices, and other technology-driven services in medicine and healthcare is leading to the generation and availability of new types of data collected during the routine delivery of health care, commonly referred to as “real-world data” or “RWD” (21;22). The sources of this RWD include electronic health records, medical claims and billing data, product and disease registries, and health administrative data.

In turn, the diversification of data sources is accompanied by an increasing variety of methods to make use of RWD and generate RWE (22), including, for example, pragmatic clinical trials, and different studies based on the applications of machine learning (ML) and artificial intelligence (AI) techniques (21).

All of this is contributing to the emergence of a new paradigm for the synthesis of evidence that goes beyond the two traditional approaches, namely systematic reviews and meta-analyses (20). This is the so-called “living” paradigm, which essentially refers to the methodological approach – elaborated upon in the last decade but difficult to put into practice until now (23) – that allows new research findings to be continually incorporated into evidence synthesis as they become available (20). Examples of living systematic reviews (such as COVID-19 or COVID-NMA), of living meta-analyses (like metaCOVID), or of projects (like More-EUROPA 2023–2027) are already well established. *Living HTA* – that is an HTA that is “planned from the outset to be updated at regular intervals or at specific trigger points (e.g., in light of new evidence and/or feedback from stakeholders)” – is an emerging concept in the field of HTA as well (24).

The latest frontier in the field of evidence synthesis is represented by the use of machine automation or, at least, by the integration of human effort and ML. In fact, recent advances in natural language processing, text mining, and ML are producing algorithms that can accurately mimic human effort in systematic review activity in a faster and cheaper manner (25). This integration can be used at various stages within the evidence review pathway: for example, it can assist in creating more effective search strategies or prioritizing articles for automatic screening and information extraction. Thus, semiautomating systematic reviews via ML and natural language processing now represent a promising subfield of study (26).

What are the ethical implications of these advancements for HTA?

Firstly, even though access to new sources of data represents an opportunity to meaningfully incorporate information beyond that drawn from published articles, managing multiple, scattered, and big data extracted from different sources may increase (or decrease) the probability of both errors and uncertainties in HTA. In other words, it could be more challenging to assess the potential benefits and harms of a technology using vast amounts of evidence of different types that come from diverse sources and may conflict. As a result, the evaluation of a specific technology may be mistaken (error), concluding that a technology has added value when it has not, or concluding that it has no added value when it has. If we make such mistakes, various parties are harmed in a variety of ways (patients not getting access to a beneficial technology, or patients getting access to a technology that is not beneficial but possibly harmful, communities bearing costs of technologies that are of low value, etc).

Secondly, as the amount of data increases, their access and availability can vary significantly across countries. Pongiglione et al. (27) mapped existing data sources in Europe for three case studies: hip and knee arthroplasty, transcatheter aortic valve implantation and transcatheter mitral valve repair, and robotic surgery procedures. They showed that the amount, content, and quality of data sources varied dramatically across countries. Even though HTA is always context-dependent, disparities in terms of data access and availability may produce evaluations that are more or less accurate and reliable. Again, if an evaluation is less accurate and reliable, various parties could be harmed in a variety of ways, for example, by not adopting effective technologies and adopting ineffective ones. Furthermore, this could result in inequalities among the various HTA agencies, with agencies being grouped based on data they are able to access.

Thirdly, evidence produced by means other than RCTs is likely to suffer more from confounding and various types of bias – a claim that is supported by both theory and empirical findings and has been at the center of a vast debate (28). As such, any judgments and decisions made on the basis of non-RCT evidence of effectiveness are likely to be more prone to error. Then, the question is whether the use of non-RCT evidence of effectiveness can be justified on the basis of other ethical considerations. That might be the case, for instance, when RCTs are considered not feasible, as in the case of rare diseases. How might such conflict be resolved?

Fourthly, systematic reviews and meta-analyses are a trusted source of information for policy development (29). Once automation is applied, the “explicability” of evidence synthesis may be difficult to ascertain. Explainable artificial intelligence (EAI) is a set of processes and methods which are intended to allow human users to understand and trust the results and output created by algorithms. Nonetheless, EAI may still contain “epistemic opaqueness,” meaning that the algorithms include elements which a human user does not or even cannot understand. Therefore, semiautomating systematic reviews may raise concerns regarding the transparency and comprehensibility of the algorithms used by the tools and, consequently, undermine trust in this type of technology.

Finally, new data sources, study designs, and technological instruments may raise questions regarding comprehension overall. Does the HTA producer have sufficient expertise to understand and skills to navigate the new “world” of evidence? Many of the innovations are unfamiliar to HTA researchers and require significant skills and expertise in handling data formats, managing deployment processes (including the use of data-sharing platforms), and so on. Similar skills are paramount to ensure that information is properly interpreted, synthetized, and above all used. Consequently, HTA researchers need to receive training and education to appropriately conduct HTA in this new landscape.

## Shifting standards of evidence

When making decisions about the approval of medicines (or other technologies), regulatory bodies such as the European Medicines Agency (EMA) or the U.S. Food and Drug Administration (FDA) agree that what matters for regulatory purposes is a medicine’s absolute efficacy and safety. By considering these characteristics, they express their commitment to the values of beneficence (acting to provide benefit to the patient) and nonmaleficence (refraining from harming the patient). Following market approval, HTA bodies, such as England’s National Institute for Health and Care Excellence (NICE) or Canada’s Drug and Health Technology Agency (CADTH), commit themselves to the same values (beneficence and nonmaleficence), albeit in a quite different way (relative effectiveness *versus* absolute efficacy), as a newly approved medicine is expected to either be more beneficial (more efficacious or safer) *compared to* the standard of care, or, at least, serve as a therapeutic alternative (have a positive benefit–harm ratio).

In addition to those values, the HTA bodies also often need to answer the question of affordability, which expresses a commitment to the value of distributive justice (the fair distribution of limited resources). Normative judgements about whether to reimburse a health technology such as a drug do not only involve a consideration of the relevant values but also require judgments of what can be considered good empirical information (evidence) to assess the extent to which those values are being accounted for or challenged by a reimbursement decision.

To provide patients with early access to promising new treatments, regulatory bodies are making decisions regarding the level of evidence required for marketing approval (13). As mentioned above, regulatory and reimbursement processes should be closely aligned, so changes in the evidence requirements by regulatory agencies put “pressure” on HTA bodies to accept similar changes in evidence requirements (see examples in Table 1). Changes in the quality of the empirical evidence have important ethical implications such resource allocation decisions, credibility of an HTA agency, and, more generally, trust in HTA as a decision-making tool.

**Table 1.** Examples of deviations in traditional standards of evidence accepted by regulatory agencies

Population	Clinical trial used for approval was not of a sufficient size (“powered”) to confidently answer the reimbursement question pertaining to a specific population or subgroup
Intervention	Intervention may require a concomitant therapy not presently reimbursed in the respective healthcare system
Comparators	Comparators may not reflect the replaced therapies in the clinical practice or may not be present at all if single-arm trials are accepted as the evidence for approval
Outcomes	Benefit in surrogate outcomes may be used for approval, creating uncertainty in HTA stemming from not using primary outcomes in economic modelling

If HTA bodies are to fulfil their ethical obligations to health care decision-makers and the population they serve, these bodies should make all challenges they face in fulfilling their functions (e.g. scientific and ethical) transparent.

Finding solutions to these challenges is usually outside the scope of HTA bodies, though they may, depending on their remit, suggest, for instance, a limited reimbursement period contingent upon a future reassessment (30), which accepts a higher risk of harm to patients to gather information to determine the (cost-)effectiveness of the relevant technology under consideration. HTA bodies are, however, reluctant to increase the probability of harm to patients in order to collect additional evidence of effectiveness and may prefer to engage in joint scientific consultation or scientific advice preapproval to strive for greater alignment between the marketing approval and reimbursement (31).

Gathering credible evidence for making ethically justified resource allocation decisions is challenging. HTAs for complex technologies which are already challenging, such as treatments for rare and life-threatening diseases, are made even more challenging by other ethical values that are put forward by patients, clinicians, and manufacturers, such as hope (whether this is rightly considered a value is not agreed by ethicists (32)) and the “right to try” novel therapies, for which harm may be greater than the benefit, as well as compassionate use, which is motivated by a persistent and great unmet need.

The increasing divergence between regulatory and HTA questions (and the empirical basis being used to answer them) may pose additional ethical problems related to the institutional integrity and trustworthiness of HTA. For example, Ataluren for treating patients with Duchenne muscular dystrophy, particularly patients with a genetic defect called a “nonsense mutation” in the dystrophin gene (33), received conditional approval from EMA in July 2014 and was subject to annual renewals based on the results of additional studies. In 2016, EMA’s Committee for Medicinal Products for Human Use (CHMP) requested a new study on Ataluren’s efficacy. In the subgroup of patients for which the medicine was considered, Ataluren did not show a statistically significant effect when compared to placebo in the primary outcome of distance walked in 6 min after 18 months. The new study conducted on a broader population failed to confirm the effect seen in the initial study of Ataluren, and this finding was corroborated by a registry analysis that had more than a 5-yr follow-up. EMA’s CHMP concluded that the initial effect observed in a smaller sample of patients was due to chance. Based on the new evidence, CHMP concluded that the benefit–risk ratio of Ataluren was negative and decided not to renew its marketing authorization. The challenge for HTA bodies lies in making a potential negative reimbursement decision after a period of conditional marketing approval of Ataluren in 2014 and the present nonrenewal decision by EMA in 2023. As the initial approval was based on the hope that Ataluren’s benefit would be proven in time, HTA bodies had to assess the size of the clinical benefit and the related price under significant uncertainty. When Ataluren’s benefit was not confirmed, the trustworthiness of the HTA agency’s conclusions was put into question.

## Discussion

We have pointed to a range of epistemic challenges with evidence production that raise ethical issues in general and for HTA in particular. Some of the issues are related to new methods of evidence production and collection, and some to evidence standards. In both cases, we are faced with two basic questions: 1) Is the evidence production and management aligned with the basic goals of medicine? 2) How much uncertainty are we willing to accept in order to potentially do good, i.e., how much positive or negative value does uncertainty have compared to the potential good or bad outcome?

These are basic questions that have haunted medicine since Hippocrates raised the critical question “how do we know that it works?” and will clearly not be answered in this article. However, we have tried to highlight that what look like methodological questions within scientific evidence production and HTA are contingent on – or perhaps raise again (34) – more overarching ethical issues related to the goals of medicine, i.e., to relieve from pain and suffering, to promote health, or to prevent disease.

The increased number of data sources and assistive technologies for knowledge analysis and synthesis enhances a traditional challenge in all evidence production, i.e., validation. The same goes for the many new study designs that facilitate evidence production in new areas (e.g., due to limited times or restricted number of patients to include) (35;36). In addition to the question of evidence validation, the issue of trustworthiness of evidence-assessing agencies (HTA) and regulatory bodies is at the fore in evidence standard setting (37).

The ethical imperative of helping persons in an effective, safe, and equitable manner urges us to improve the ways we produce and evaluate evidence. Hence, new modes of evidence production are welcome. However, there is no doubt that several (commercial) actors have strong interests in lowering the standards of evidence requirements and pushing more of the evidence production costs (directly) to the patients and payers (38;39).

While it is urgent to provide efficient and safe health services to patients as soon as possible, it is equally important not to start and continue to provide services that are inefficient and/or harmful. With health technologies, beginnings count (40) as there is a technological imperative (41;42) and reversal and deimplementation are demonstrated to be very difficult (43).

In the European context, many of the challenges we have identified may perhaps find a solution through the implementation from 2025 of the new EU HTA Regulation (EU) 2021/2282, which focuses on aligning regulatory agencies with HTA bodies, providing for centralized joint clinical assessment (44).

## Conclusion

In this article, we have identified some emerging trends in HTA that raise ethical issues in general and for HTA in particular. Although some of the issues are related to new modes of evidence production and collection, others are connected to changing evidence standards used in regulatory and reimbursement environments. The intention of this article has mainly been to lay the groundwork for a more detailed discussion, as there are many more issues to address.

Common to all identified issues is how to maintain trustworthiness, trust in HTA, and trust in the healthcare system more broadly. To find good ways of providing robust evidence for effective and sustainable health care, we need to pay particular attention to fair, explicit, and transparent processes.

## Supporting information

Refolo et al. supplementary material Refolo et al. supplementary material

## Supplementary material

The supplementary material for this article can be found at http://doi.org/10.1017/S0266462324000394.
