# Supplementary material for: Ethical challenges for Health Technology Assessment (HTA) in the evolving evidence landscape
Source: Int J Technol Assess Health Care. 2024 Nov 4;40(1):e39. doi: 10.1017/S0266462324000394 (PMC11569911; doi:10.1017/S0266462324000394)
Supplement: Refolo et al. supplementary material [file S0266462324000394sup001.docx]

**List of participants**

The first meeting was held online on March 9, 2023. The list of participants is the following: Ken Bond, Katherine Duthie, Björn Hofmann, Elena Petelos, Costanza Raiomondi Michal Stanak, Despina Voulgaraki.

The face-to-face meeting was held in Rome, Italy, on March 30-April 1, 2023. The list of participants is the following: Neil Bertelsen, Ken Bond, Katherine Duthie, Björn Hofmann, Costanza Raiomondi Michal Stanak, Despina Voulgaraki.
